# *Toxocara canis* Differentially Affects Hepatic MicroRNA Expression in Beagle Dogs at Different Stages of Infection

**DOI:** 10.3389/fvets.2020.587273

**Published:** 2020-11-12

**Authors:** Yang Zou, Wen-Bin Zheng, Jun-Jun He, Hany M. Elsheikha, Xing-Quan Zhu, Yi-Xin Lu

**Affiliations:** ^1^Heilongjiang Key Laboratory for Zoonosis, College of Veterinary Medicine, Northeast Agricultural University, Harbin, China; ^2^State Key Laboratory of Veterinary Etiological Biology, Key Laboratory of Veterinary Parasitology of Gansu Province, Lanzhou Veterinary Research Institute, Chinese Academy of Agricultural Sciences, Lanzhou, China; ^3^Faculty of Medicine and Health Sciences, School of Veterinary Medicine and Science, University of Nottingham, Loughborough, United Kingdom; ^4^College of Veterinary Medicine, Shanxi Agricultural University, Taigu, China

**Keywords:** *Toxocara canis*, miRNAs, beagle dogs, liver, RNA-seq

## Abstract

*Toxocara canis* is a neglected zoonotic parasite, which threatens the health of dogs and humans worldwide. The molecular mechanisms that underlie the progression of *T. canis* infection remain mostly unknown. MicroRNAs (miRNAs) are small non-coding RNAs that have been identified in *T. canis*; however, the regulation and role of miRNAs in the host during infection remain incompletely understood. In this study, we determined hepatic miRNA expression at different stages of *T. canis* infection in beagle dogs. Individual dogs were infected by 300 embryonated *T. canis* eggs, and their livers were collected at 12 hpi (hours post-infection), 24 hpi, and 36 dpi (days post-infection). The expression profiles of liver miRNAs were determined using RNA-sequencing. Compared to the control groups, 9, 16, and 34 differentially expressed miRNAs (DEmiRNAs) were detected in the livers of infected dogs at the three infection stages, respectively. Among those DEmiRNAs, the novel-294 and cfa-miR-885 were predicted to regulate inflammation-related genes at the initial stage of infection (12 hpi). The cfa-miR-1839 was predicted to regulate the target gene TRIM71, which may influence the development of *T. canis* larvae at 24 hpi. Moreover, cfa-miR-370 and cfa-miR-133c were associated with immune response at the final stage of infection (36 dpi). Some immunity-related Gene Ontology terms were enriched particularly at 24 hpi. Likewise, Kyoto Encyclopedia of Genes and Genomes pathway analysis showed that many significantly enriched pathways were involved in inflammation and immune responses. The expression level of nine DEmiRNAs was validated using quantitative real-time PCR (qRT-PCR). These results show that miRNAs play critical roles in the pathogenesis of *T. canis* during the hepatic phase of parasite development. Our data provide fundamental information for further investigation of the roles of miRNAs in the innate/adaptive immune response of dogs infected by *T. canis*.

## Introduction

Toxocariasis, mainly caused by *Toxocara canis* infection in dogs (1), is a neglected zoonosis worldwide. Dogs, as the definitive hosts of *T. canis*, can excrete eggs with feces, leading to environment contamination (2, 3). Humans can be infected by *T. canis* via ingestion of food contaminated with embryonated eggs or larvae (2, 4). The larvae hatch inside the intestinal tract of the host and then migrate to the other parts of the body, leading to serious health problems, such as visceral larva migrans, ocular larva migrans, and neurotoxocariasis (5, 6). Owing to non-specific symptoms in human infection and the diagnostic challenges (7), the public health impact caused by *T. canis* infection may be ignored. More efforts are needed to achieve better understanding of the pathogenesis of toxocariasis (1).

In recent years, multiple approaches, such as genomics, transcriptomics, and proteomics, have been applied to characterize the biological and molecular features of *T. canis* (8–11). However, there is a dearth of information regarding the interaction between *T. canis* and its host. MicroRNAs (miRNAs) are endogenous, small, non-coding RNAs that have received significant attention from the scientific community due to their involvement in many and diverse biological processes, such as apoptosis, proliferation, metabolism, and immune response (12). miRNAs together with transcription factors have been considered key regulators that modulate the expression levels of almost all genes that mediate various pathophysiological processes (13, 14). Also, parasites can alter the expression level of host miRNAs in order to regulate gene expression of the target tissues (15–17). miRNAs can repress mRNA expression through binding to the 3′ untranslated regions of target genes or by enhancing mRNA degradation (18). A previous study showed that *T. canis* miRNAs, Tc-let-7-5p, Tc-miR-34, and Tc-miR-100, play roles in host–parasite interactions (8). However, the regulation of miRNAs and their roles in the pathogenesis of *T. canis* during the hepatic phase of infection remains mostly unknown.

Therefore, the present study aimed to investigate the alteration of miRNA expression profiles in the livers of beagle dogs infected by *T. canis* at different stages of infection using small RNA transcriptome sequencing and bioinformatics analysis. The study findings revealed that some differentially expressed miRNAs (DEmiRNAs) play roles in the regulation of inflammatory and immune responses of puppies against *T. canis* infection.

## Materials and Methods

### Experimental Infection of Dogs

The adult *T. canis* worms were collected from naturally infected dogs in Rongchang District, Chongqing Municipality, China, and female adult *T. canis* were identified based on gross morphology. The eggs of *T. canis* were obtained from the uteri of female *T. canis* worms. The unembryonated eggs were incubated with 0.5% formalin solution at 28°C (85–95% humidity) for 28 days, and then the eggs were harvested and filtered through a 200-mesh screen. The finally embryonated eggs were stored in 1% formalin solution at 4°C. Eighteen beagle puppies (6–7 weeks old) were provided by the National Canine Laboratory Animal Resource Center and housed following Good Laboratory Practice (GLP) in an animal facility, according to the GB standard (GB 14922.2-2011) of China. All puppies had tested negative for *T. canis* infection by indirect ELISA, using larval ES antigen, before the start of the experiment. Feces of individual puppies were collected daily and examined using a standard sugar floatation method. Puppies were equally allocated into three experimental and three control groups (three puppies per group). The puppy groups were housed separately and were provided with similar conditions including access to the same water source and food supply. Each puppy was orally infected with 300 embryonated eggs in 1 ml normal saline solution, while the control puppies were inoculated with the same amount of saline but without any eggs.

### Detection of *T. canis* Infection

Blood samples of each puppy were collected from the jugular vein into sterile tubes containing EDTA-K2 and tubes without anticoagulant. The *T. canis* IgG antibody was detected using indirect ELISA as previously described (19). Light microscopy was also used to observe whether *T. canis* larvae are present in the livers of puppies. The genomic DNA of infectious eggs (used to infect puppies), larvae (isolated from the liver of infected puppies), and adult *T. canis* (recovered from the small intestine of infected puppies) were isolated using a DNA extraction kit (TianGen™, Beijing, China) according to the manufacturer's instruction. The isolated DNA samples were analyzed using PCR with primer sequences specific to the internal transcribed spacer (ITS) region (partial sequence of ITS-1 and ITS-2) (20). The positive PCR products were sequenced by Sangon Biotech (Shanghai, China), and the obtained sequences were searched against similar sequences available in the GenBank database using Nucleotide BLAST (https://blast.ncbi.nlm.nih.gov/Blast.cgi).

### Sample Collection, RNA Extraction, and Quantification

Infected puppies (*n* = 9) and naive (control) puppies (*n* = 9) were killed by potassium chloride (KCl) under a general anesthetic [50 mg/kg tiletamine-zolazepam (Zoletil®), Virbac, France]. The liver samples were collected from all puppies at 12 h post-infection (hpi), 24 hpi, and 36 days post-infection (dpi) according to methods described in our previous study (21). Three biological replicates were examined in each group at each time point post-infection. The collected liver samples were quickly stored in liquid nitrogen until used for RNA extraction. The larvae were recovered from the remaining fresh liver sample of each puppy using the modified Baermann funnel method as previously described (22). The total RNA was extracted from the liver samples of puppies using TRIZOL (Life Technologies, CA, USA). The genomic DNA was removed using DNase I (NEB, Ipswich, USA). RNA concentration was measured using the Qubit® RNA Assay Kit and Qubit® 2.0 Fluorometer (Life Technologies, CA, USA). The integrity of RNA was assessed by the RNA Nano 6000 Assay Kit and the Agilent Bioanalyzer 2100 system (Agilent Technologies, CA, USA). The purity of RNA was examined using the NanoPhotometer® spectrophotometer (IMPLEN, CA, USA). High-quality RNA samples with RNA integrity numbers (RINs) >8.0 were used to construct the sequencing library.

### Small RNA Library Preparation and Sequencing

A total of 3 μg RNA of each sample was used for the construction of the small RNA library by using the NEBNext® Multiplex Small RNA Library Prep Set for Illumina® (NEB, USA). The constructed libraries were sequenced on an Illumina Hiseq 2500 platform. The reads that contain poly N, 5′ adapter contaminants, without 3′ adapter or the insert tag (including poly G, C, A, or T and low-quality reads) were filtered from raw data using custom perl and python scripts. The clean reads that ranged from 18 to 35 nt were mapped against the reference sequence by Bowtie (23). The mapped sRNAs were searched against miRBase20.0 data to identify known miRNAs. In addition, mirdeep2 (24) and miREvo (25) were used to identify potential novel miRNAs. The novel miRNA was predicted by the characteristics of the hairpin structure of the miRNA precursor. The expression levels of miRNA were estimated by TPM (transcript per million) (26). Differential expression analysis was performed using the DESeq R package (1.8.3). A *P*-value < 0.05 was used as the significance threshold value of differential expression.

### Bioinformatics Analysis of DEmiRNA

The target genes of miRNAs were predicted by RNAhybrid, PITA tools, and miRanda (27). To predict the function of the target gene of DEmiRNAs, GOseq R package (28), and KOBAS software (29, 30) were used for Gene Ontology (GO) enrichment and Kyoto Encyclopedia of Genes and Genomes (KEGG) pathway analysis, respectively. A *P-*value < 0.05 was considered as significantly enriched.

### Verification of miRNA Expression by Quantitative Real-Time PCR

Quantitative real-time PCR (qRT-PCR) was performed to confirm the upregulation and downregulation of nine miRNAs randomly selected for verification of the RNA-seq results. qRT-PCR was performed using an miRcute enhanced miRNA qRT-PCR Kit (TianGen, Beijing, China) on a LightCycler480 (Roche, Basle, Switzerland). The cDNA of miRNA was synthesized using an miRcute enhanced miRNA cDNA first chain synthesis Kit (TianGen, Beijing, China). Then, 1 μl cDNA was used for qRT-PCR as follows: initial denaturation at 95°C for 15 min, followed by 40 cycles of 94°C for 20 s and 60°C for 34 s. All primers are listed in Table 1. The U6 small nuclear RNA (snRNA) was used as an internal control gene. Melting curve analysis (95°C for 10 s, 65°C for 1 min, and progressive increase from 65°C to 95°C) was performed to ensure specific amplification in each reaction. Each reaction included a blank control to rule out the presence of contamination. The relative expression quantity was analyzed by the ^2−ΔΔ^Ct method (31, 32).

**Table 1:** Primers used in microRNA (miRNA)–specific quantitative real-time PCR (qRT-PCR) analysis.

**miRNAs**	**Primer**	**Sequence (5′ to 3′)**
U6	Forward primer	CGCTTCGGCAGCACATATAC
Cfa-miR-381	Forward primer	CTGGGTCTGGTATACAAGGGCAAGCTCTC
Cfa-miR-10b	Forward primer	CTGGGTCTGGTATACAAGGGCAAGCTCTC
Cfa-miR-194	Forward primer	CTGGGTCTGGTGTAACAGCAACTCCATGT
Cfa-miR-125a	Forward primer	CTGGGTCTGGTCCCTGAGACCCTTTAAC
Cfa-miR-371	Forward primer	CTGGGTCTGGACTCAAAAAATGGCGGCA
Cfa-miR-16	Forward primer	CTGGGTCTGGTAGCAGCACGTAAATATTGG
Cfa-miR-10a	Forward primer	CTGGGTCTGGTACCCTGTAGATCCGAA
Cfa-miR-146a	Forward primer	CTGGGTCTGGTGAGAACTGAATTCCATGGG

## Results

### Detection of *T. canis* Infection in Beagle Dogs

A blood sample of each puppy was collected and tested for IgG antibody against *T. canis*. The IgG antibodies of *T. canis* were detected in infected puppies at 36 dpi. At 12 hpi, *T. canis* larvae were recovered from the livers of three infected puppies, and at 24 hpi, *T. canis* larvae were recovered from the livers of all infected puppies. At 36 dpi, *T. canis* larvae were detected in the liver of one infected puppy, and adult *T. canis* were recovered in the small intestine of all infected puppies. Furthermore, no *T. canis* larvae and anti–*T. canis* IgG antibodies were found in the control puppies. The sequences obtained from the embryonated eggs, larvae, and adult worms were found to match the sequence of *T. canis* (GenBank Accession No. JF837169.1).

### Characteristics of the Sequenced Data

In each miRNA library, 142,605,411 raw reads and 7.131 Gb raw data were obtained from infected puppy groups, whereas 136,764,149 raw reads and 6.898 Gb raw data were obtained from the control groups. More than 99% of reads had sequencing quality >Q20 (Table 2). The clean reads with appropriate 18–35 nt lengths were selected for further analysis. Moreover, 52.72–65.17 and 0.17–0.24% unique reads were confirmed as known and novel miRNAs, respectively. In addition, 22.16–29.70% non-annotated reads were found in this study (Supplementary Table 1), suggesting that these non- annotated reads could be involved in the pathogenesis and progression of *T. canis* infection.

**Table 2:** Summary of the quality control parameters of the reads.

**Groups**	**Samples**	**Raw reads**	**Clean reads**	**Bases**	**Error rate (%)**	**Q20 (%)**	**Q30 (%)**	**GC content (%)**
12 hpi	A12hT1	17,052,302	16,734,756	0.853G	0.01	99.72	99.28	49.61
	A12hT2	15,050,522	14,632,243	0.753G	0.01	99.69	99.24	49.39
	A12hT3	15,753,780	15,606,433	0.788G	0.01	99.70	99.21	49.92
	A12hC1	18,499,157	18,320,368	0.925G	0.01	99.72	99.27	49.75
	A12hC2	15,502,426	15,346,075	0.775G	0.01	99.64	99.09	49.73
	A12hC3	13,471,115	13,320,106	0.674G	0.01	99.65	99.13	49.71
24 hpi	B24hT1	16,220,657	16,074,374	0.811G	0.01	99.57	98.92	49.48
	B24hT2	14,587,167	14,466,523	0.729G	0.01	99.69	99.20	49.60
	B24hT3	17,146,312	16,980,582	0.857G	0.01	99.62	99.04	49.76
	B24hC1	14,239,211	14,125,501	0.712G	0.01	99.64	99.10	49.01
	B24hC2	15,619,834	15,495,519	0.781G	0.01	99.58	98.97	49.15
	B24hC3	14,913,621	14,810,484	0.746G	0.01	99.70	99.23	49.20
36 dpi	D36dT1	18,000,592	17,826,356	0.900G	0.01	99.57	98.93	49.39
	D36dT2	15,372,801	15,223,534	0.769G	0.01	99.72	99.26	49.52
	D36dT3	13,421,278	13,326,515	0.671G	0.01	99.79	99.45	49.33
	D36dC1	13,738,157	13,648,316	0.687G	0.01	99.73	99.31	49.23
	D36dC2	15,012,200	14,900,388	0.751G	0.01	99.73	99.32	49.51
	D36dC3	16,947,963	16,797,392	0.847G	0.01	99.75	99.36	49.26

### Differentially Expressed Hepatic miRNAs (DEmiRNAs) at Different Infection stages

**Figure 1:**
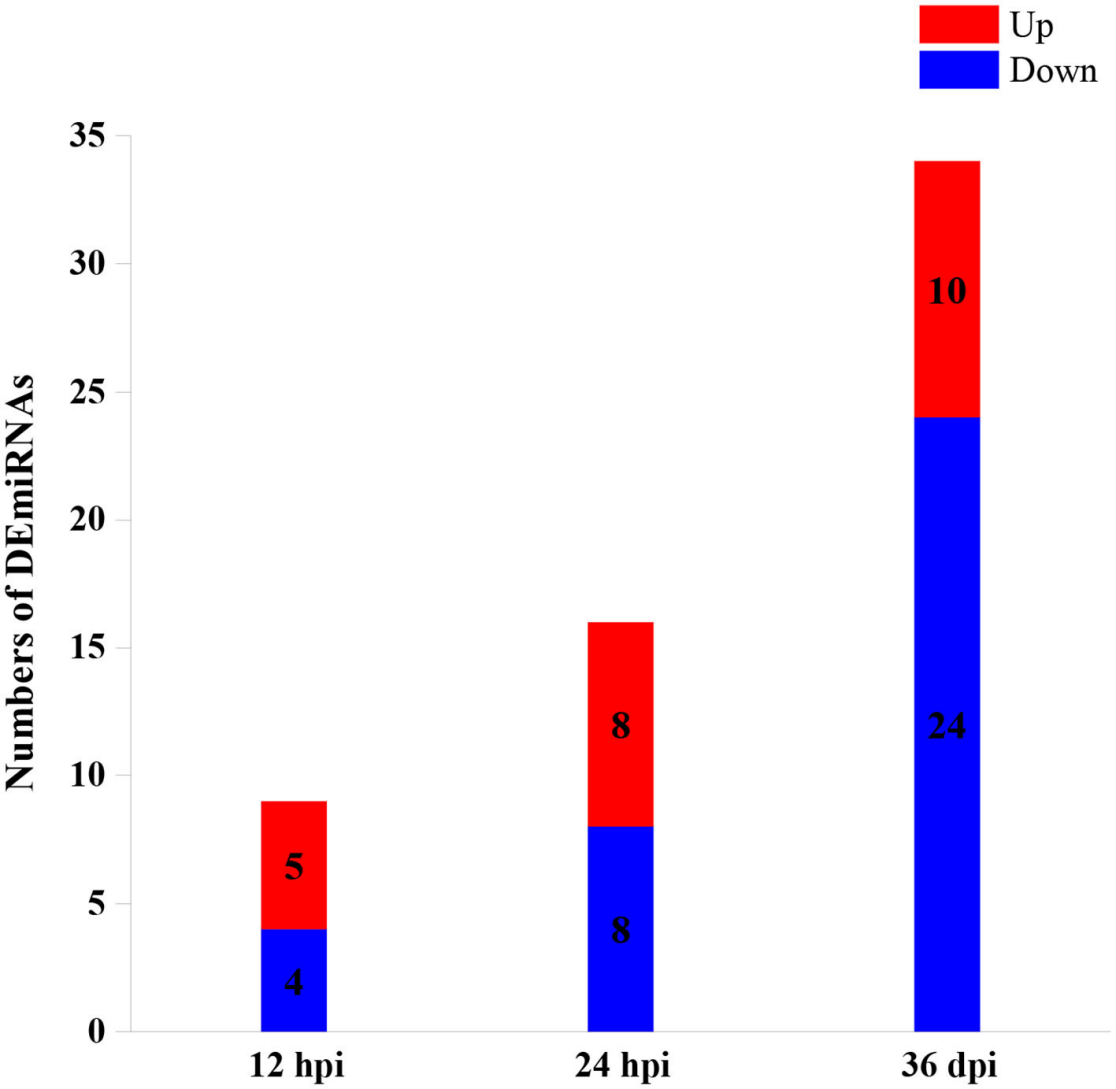
The numbers of differentially expressed microRNAs (DEmiRNAs) at three infection stages [12 h post-infection (hpi), 24 hpi, and 36 days post-infection (dpi)]. The red and blue colors represent the upregulated and downregulated miRNAs, respectively.

**Figure 2:**
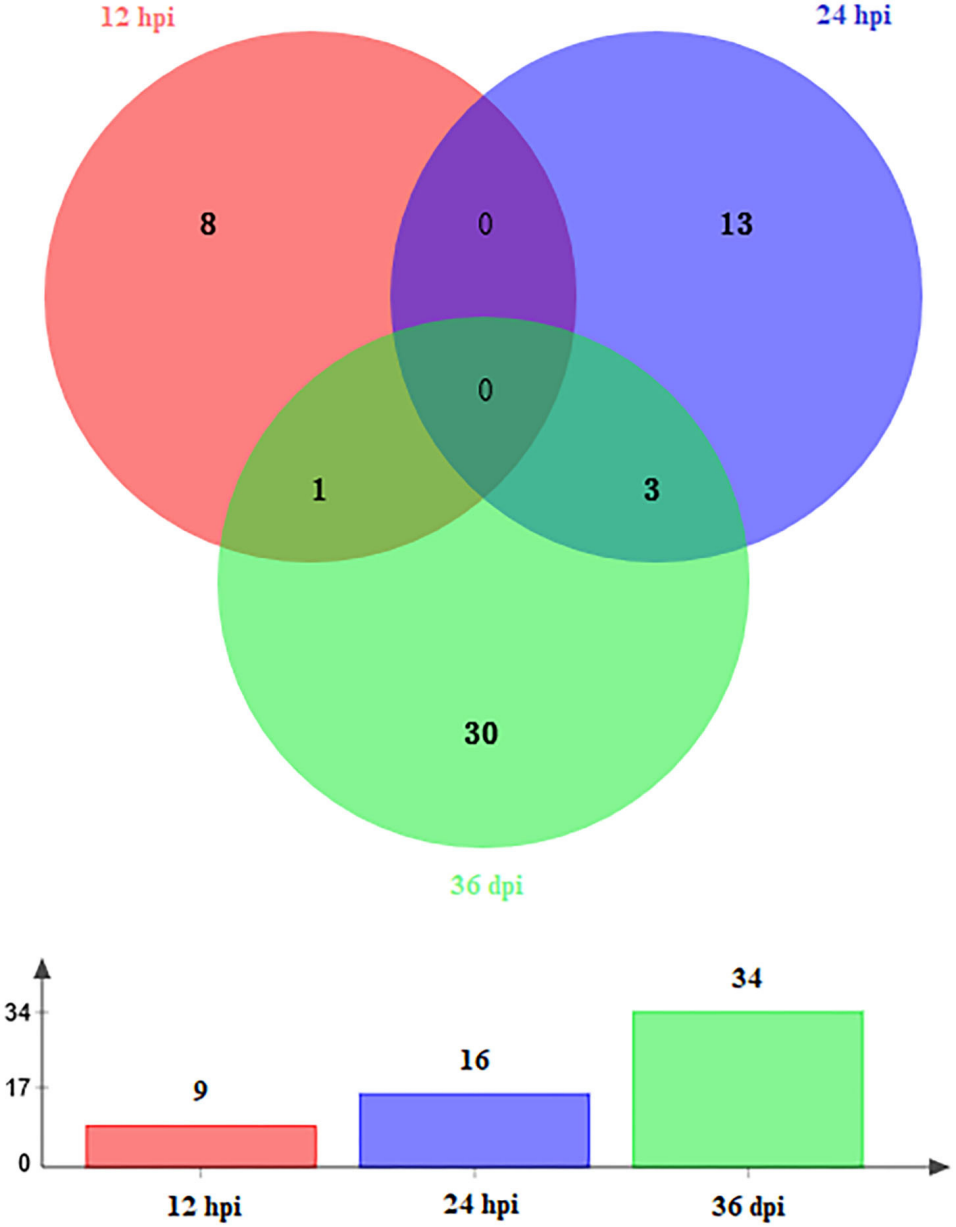
Venn diagram showing the shared and unique DEmiRNAs at different time points after infection (12 hpi, 24 hpi, and 36 dpi).

A total of 59 DEmiRNAs were identified at three infection stages, including 9, 16, and 34 DEmiRNAs at 12 hpi, 24 hpi, and 36 dpi, respectively. Details of DEmiRNAs are shown in Supplementary Table 2. Among these DEmiRNAs, 23 DEmiRNAs were upregulated, whereas 36 DEmiRNAs were downregulated (Figure 1). However, no common miRNA was found at the three infection stages (Figure 2).

### Target Gene Prediction and Functional Analysis

**Figure 3:**
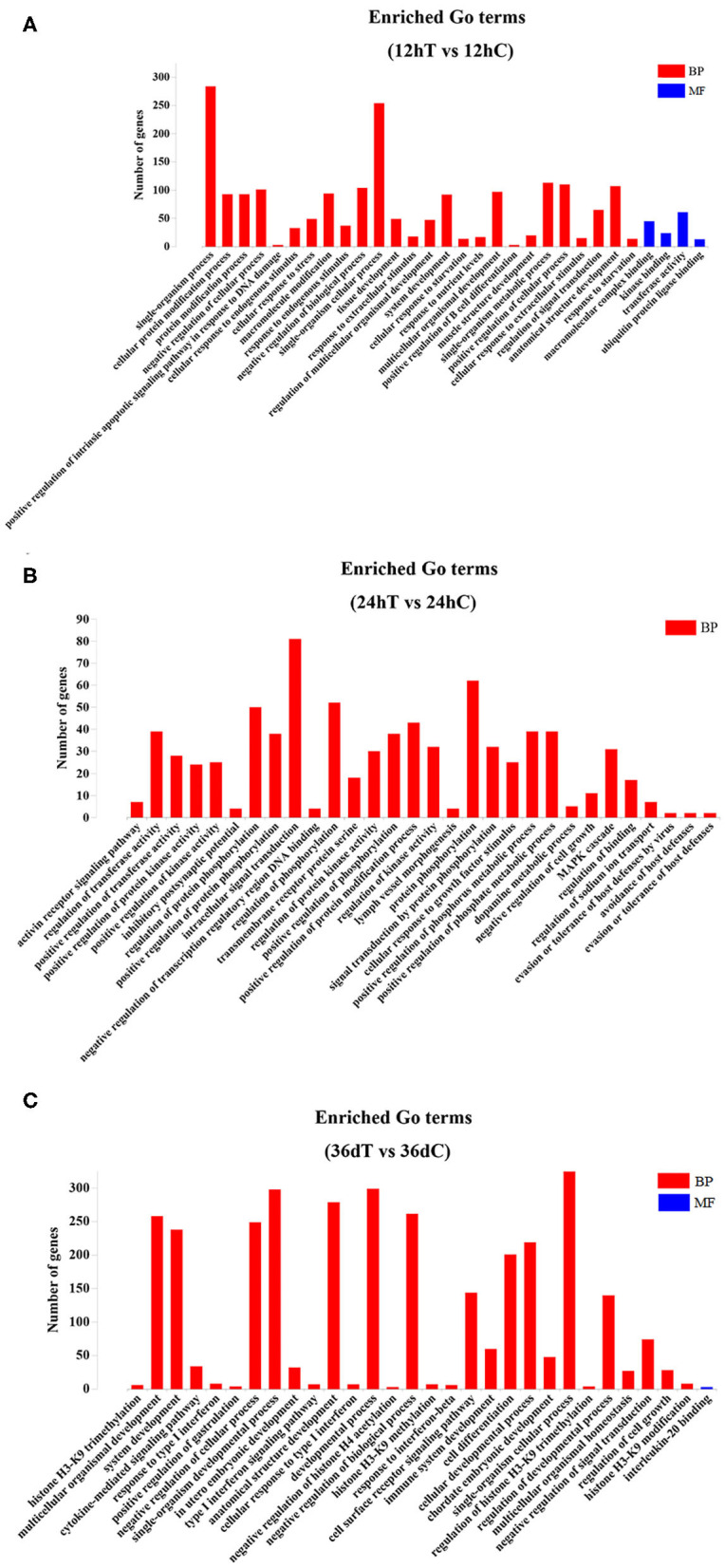
The top 30 Gene Ontology (GO) terms of the DEmiRNAs. **(A)** The significantly enriched biological process (BP) and molecular function (MF) terms of the target genes of DEmiRNAs at 12 hpi. **(B)** The significantly enriched BP terms of target genes of DEmiRNAs at 24 hpi. **(C)** The significantly enriched BP and MF terms of target genes of DEmiRNAs at 36 dpi.

Candidate target genes of DEmiRNAs of beagle dog livers were predicted by RNAhybrid, miRanda, and PITA tools. The prediction results showed that a total of 384, 518, and 1,225 hepatic genes were targeted by DEmiRNAs at 12 hpi, 24 hpi, and 36 dpi, respectively. The immunoglobulin superfamily (IgSF) is a molecular superfamily with an immunoglobulin-like domain, and most members of IgSF are found on the surface of lymphocytes and participate in various immune activities. In this study, the IgSF genes were predicted to be regulated by some miRNAs, such as novel-294, cfa-miR-25, cfa-miR-15b, cfa-miR-145, cfa-miR-150, cfa-miR-497, cfa-miR-1839 and cfa-miR-151, cfa-miR-133c, novel-337, cfa-miR-127, cfa-miR-205, and cfa-miR-194 (Supplementary Table 3). The GO enrichment analysis showed that 9 DEmiRNAs were significantly enriched in 797 GO terms at 12 hpi; 16 DEmiRNAs were significantly enriched in 768 GO terms at 24 hpi; and 34 DEmiRNAs were significantly enriched in 665 GO terms at 36 dpi (*P* < 0.05) (Supplementary Table 4). The top 30 most significant GO terms (*P* < 0.05) belonging to biological process and molecular function at each time point are shown in Figure 3. Among of these GO terms, the immune-related GO terms were found in infected livers (Supplementary Table 5). At 12 hpi, one DEmiRNA was related to three immune-related GO terms, including natural killer (NK) cell differentiation involved in immune response (GO:0002325), regulation of NK cell differentiation involved in immune response (GO:0032826), and negative regulation of NK cell differentiation involved in immune response (GO:0032827). At 24 hpi, six DEmiRNAs were significantly related to 32 immune-related GO terms, including positive regulation of B-cell–mediated immunity (GO:0002714), positive regulation of immunoglobulin-mediated immune response (GO:0002891), positive regulation of leukocyte-mediated immunity (GO:0002705), positive regulation of lymphocyte-mediated immunity (GO:0002708), and regulation of innate immune response (GO:0045088). At 36 dpi, 12 DEmiRNAs were significantly related to four immune-related GO terms, including immune system development (GO:0002520), negative regulation of innate immune response (GO:0045824), positive regulation of immune system process (GO:0002684), and negative regulation of immune response (GO:0050777) (Supplementary Table 5).

### KEGG Pathway Analysis

**Figure 4:**
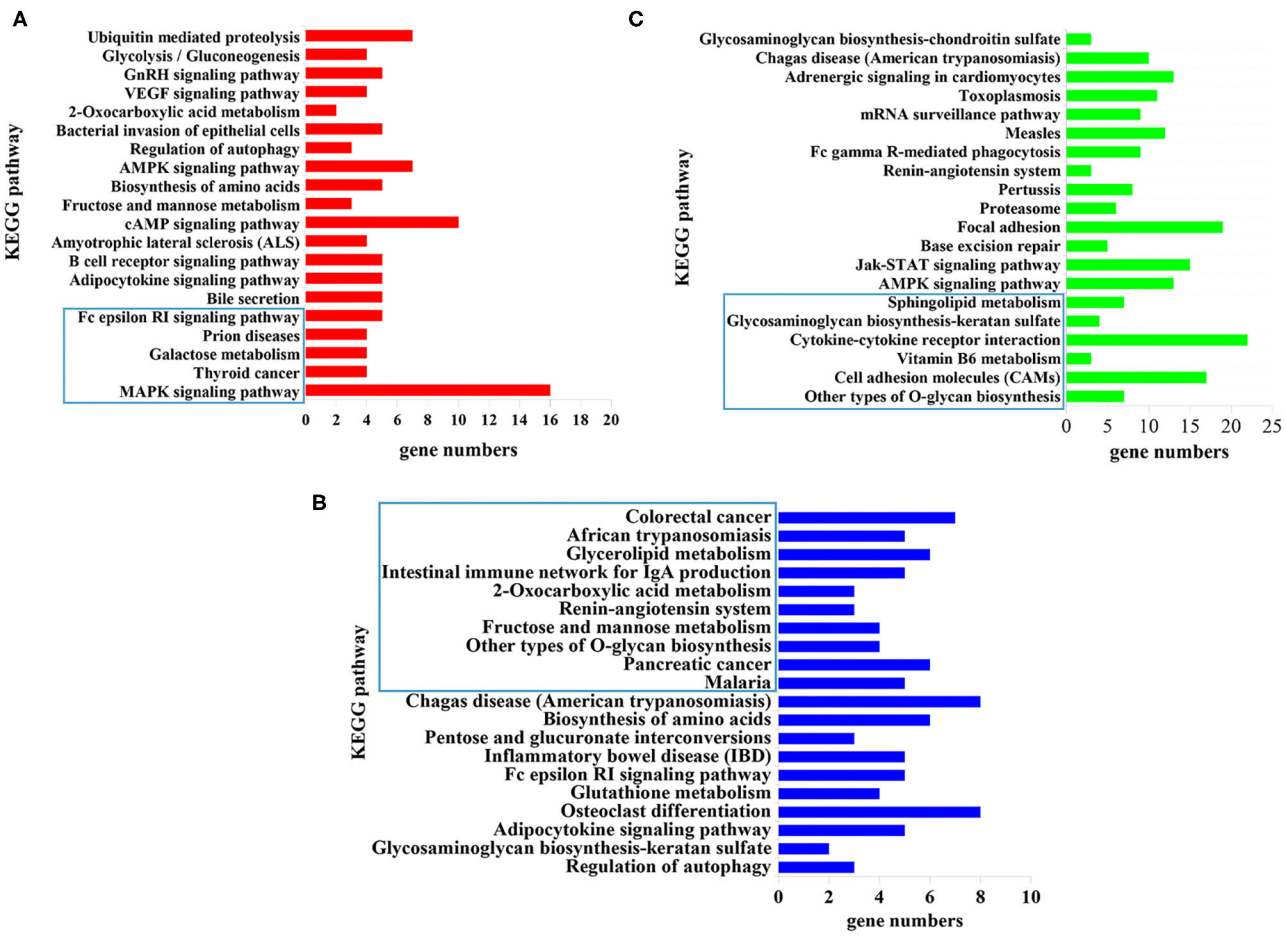
The top 20 signaling pathways enriched by the target genes of differently expressed microRNAs (miRNAs) at **(A)** 12 hpi, **(B)** 24 hpi, and **(C)** 36 dpi. The X-axis labels denote the gene numbers, and the Y-axis labels represent the names of Kyoto Encyclopedia of Genes and Genomes (KEGG) pathways. The pathways enclosed within the blue rectangle represent the significantly enriched pathways.

KEGG pathway analysis revealed that 5, 10, and 6 pathways were significantly enriched (*P* < 0.05) at 12 hpi, 24 hpi, and 36 dpi, respectively (Supplementary Table 6). The top 20 targeted pathways of DEmiRNAs are shown in Figure 4. At 12 hpi, some immune-related pathways were significantly enriched, including the mitogen-activated protein kinase (MAPK) signaling pathway (cfa04010) and Fc epsilon RI signaling pathway (cfa04664) (Figure 4A). The highly enriched pathways at 24 hpi (Figure 4B) included glycerolipid metabolism (cfa00561), the renin–angiotensin system pathway (cfa04614), and other types of O-glycan biosynthesis (cfa00514). Moreover, significantly inflammation-related pathways were found at 36 dpi, including cell adhesion molecules (CAMs) (cfa04514), vitamin B6 metabolism (cfa00750), cytokine–cytokine receptor interaction (cfa04060), and glycosaminoglycan biosynthesis–keratan sulfate (cfa00533) (Figure 4C).

### qRT-PCR Validation of RNA-Sequencing Data

**Figure 5:**
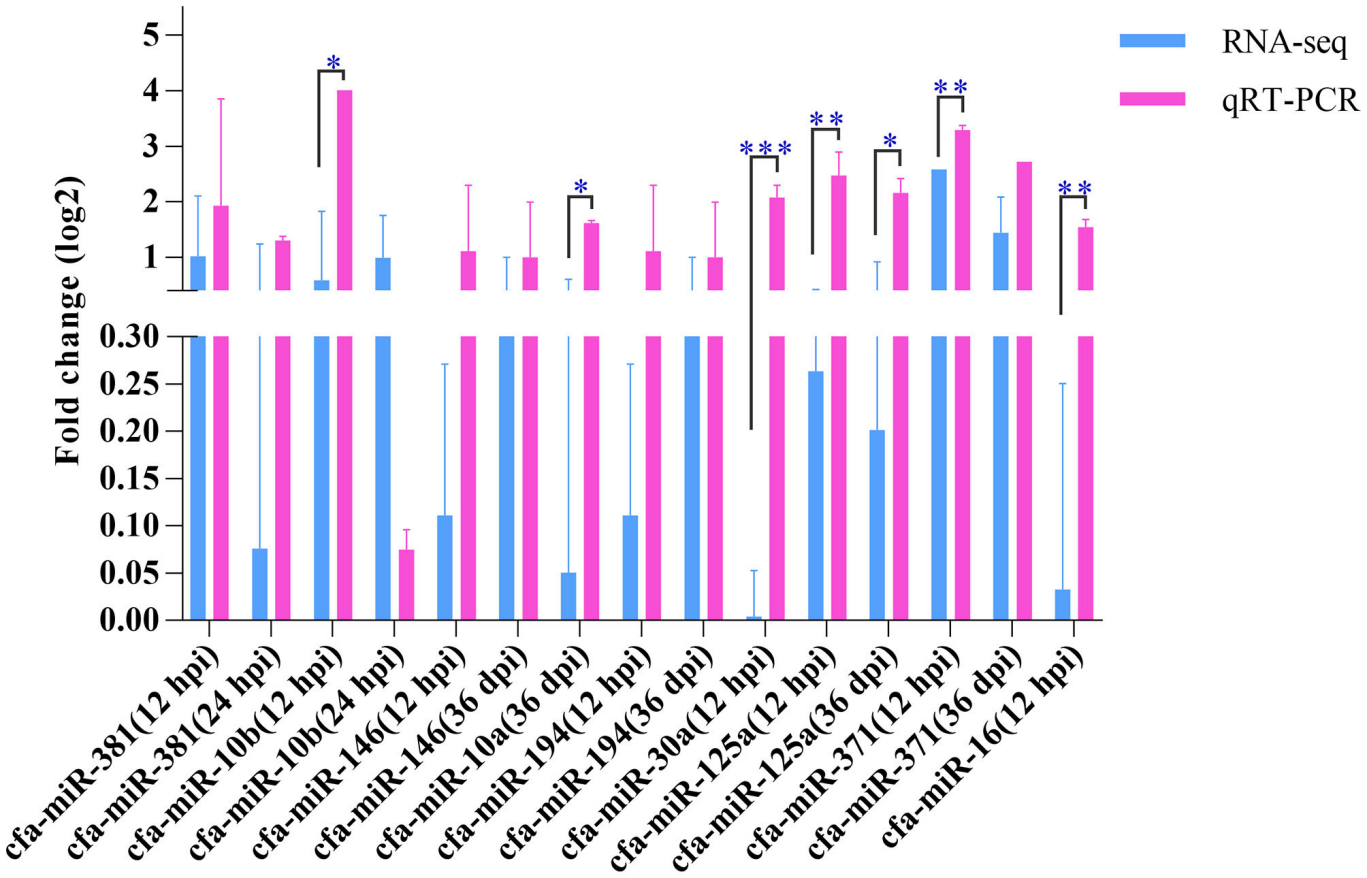
Quantitative real-time PCR. The Y-axis shows the relative change of miRNA levels expressed as fold increase compared with the control U6 small nuclear RNA (snRNA). The X-axis shows the name of the nine miRNAs used in the cross-validation of expression analysis. *P < 0.05; **P < 0.01; ***P < 0.001.

Nine miRNAs were randomly selected for qRT-PCR verification (Figure 5), including cfa-miR-381, cfa-miR-10b, cfa-miR-146, cfa-miR-10a, cfa-miR-194, cfa-miR-30a, cfa-miR-125a, cfa-miR-371, and cfa-miR-16. Although the expression levels of the nine miRNAs obtained by qRT-PCR were slightly higher those obtained by RNA-sequencing, the expression trends obtained by both methods were consistent, which suggested an increased expression of the examined miRNAs.

## Discussion

In this study, hepatic miRNA expression patterns of beagle dogs during *T. canis* infection were investigated using RNA-sequencing analysis. A total of 59 DEmiRNAs were identified, including 9, 16, and 34 DEmiRNAs at 12 hpi, 24 hpi, and 36 dpi, respectively. Most of the downregulated DEmiRNAs were found in the infected group at 36 dpi (Figure 1). This finding suggested that miRNAs were negatively regulated to alter the expression of the target genes during the later stage of infection in infected puppies' livers. Moreover, among the dysregulated miRNAs, only one and three were common between 12 hpi and 36 dpi and between 24 hpi and 36 dpi, respectively (Figure 2). This finding indicates that the expression of hepatic miRNAs of beagle dogs evolves during the *T. canis* infection. Two important miRNAs, novel-294 and cfa-miR-885, were differentially expressed at the initial stage of infection (12 hpi) (Supplementary Table 2). Furthermore, the IgSF gene *FGFR1* was predicted to be regulated by miRNA novel-294 at 12 hpi (Supplementary Table 3). A previous study found that *FGFR1* expression improved beta-cell survival in cytokine-induced inflammation (33). Hence, we speculated that the upregulation of the novel-294 in puppies' livers promotes the immune response of puppies to *T. canis* infection *via* increasing the expression of *FGFR1*. miRNA cfa-miR-885 was predicted to regulate *IGSF3* gene of the IgSF (Supplementary Table 3). The IgSF and leukocyte integrins play an important role in the regulation of leukocyte recruitment to the inflammation sites (34). These findings suggest that cfa-miR-885 plays a protective role during *T. canis* infection.

At 24 hpi, 16 miRNAs were found differentially expressed in infected livers (e.g., cfa-miR-1839). The cfa-miR-1839 was downregulated at 24 hpi, and TRIM71, the predicted target gene of cfa-miR-1839 (Supplementary Table 3), regulates juvenile-to-adult transition events in nematodes and mammals (35). The majority of *T. canis* larvae reach the liver at 24 hpi (36); subsequently, the larvae migrate to lung, muscle, and brain tissue *via* the circulation (36). However, some larvae cannot continue to migrate and are trapped in the hepatic capillaries (36). Thus, we assumed that the downregulation of hepatic cfa-miR-1839 possibly regulates the juvenile-to-adult development of *T. canis* larvae in the puppies' livers. Whether cfa-miR-1839 regulates juvenile-to-adult transition of *T. canis* larvae remains to be further investigated.

cfa-miR-370 and cfa-miR-133c were significantly downregulated at 36 dpi (Supplementary Table 2). The target gene prediction showed that cfa-miR-370 targets the *CD3E* gene (Supplementary Table 3). *CD3E*, the T-cell antigen receptor epsilon subunit gene, is essential for TCR signaling and T-cell differentiation (37, 38). In our study, cfa-miR-370 was downregulated in the infected livers at 36 dpi, indicating that the downregulation of cfa-miR-370 may represent an immunoreaction to resist *T. canis* infection. In addition, two differently expressed miRNAs, cfa-miR-370 and cfa-miR-133c, were predicted to regulate the *ARRDC1* gene (Supplementary Table 3). Additionally, cfa-miR-133c was predicted to regulate the *IL18R1* gene. Previous research found that *IL-18R*/*MyD88* plays a crucial role in the development of a robust Th1 response during *Trypanosoma cruzi* infection (39). Therefore, the abnormal expression of cfa-miR-133c seems to be a potential candidate for further study of the role of cfa-miR-133c in promoting a protective Th1 immune response to *T. canis* infection.

GO enrichment analysis showed that 797, 769, and 665 GO terms were significantly enriched at 12 hpi, 24 hpi, and 36 dpi, respectively (Supplementary Table 4). Based on the analysis of the 797 significantly enriched GO terms at 12 hpi, nine DEmiRNAs were mainly involved in the signal-related biological process, including instance single-organism processes, single-organism cellular process, and single-organism metabolic process (Figure 3A). These processes are involved in acute liver injury (40). At 24 hpi, 16 DEmiRNAs were mainly associated with biological processes, such as protein phosphorylation, intracellular signal transduction, regulation of phosphorylation, and regulation of protein phosphorylation (Figure 3B). Some phosphorylated proteins were related to TNF-α signaling (41), which regulate immune response (42). At 36 dpi, 10 upregulated and 24 downregulated miRNAs were involved in response to stress, positive regulation of the immune system process, and regulation of nervous system development (Figure 3C). Moreover, a total of 39 immunization-related GO terms were significantly enriched at three time points (Supplementary Table 5). These results show that *T. canis* larvae can elicit a significant immune response after infecting the liver.

The top 20 enriched pathways are shown in Figure 4. The target genes of the DEmiRNAs were significantly enriched in the MAPK signaling pathway, galactose metabolism, and Fc epsilon RI signaling pathway at 12 hpi (Figure 4A). The MAPK signaling pathway participated in diverse cellular responses, such as inflammation, differentiation, proliferation, and apoptosis (43). Additionally, the MAPK signaling pathway is involved in the development, reproduction, and survival of *Schistosoma mansoni* (44). The Fc epsilon RI is the primary receptor in mast cells, which can influence the synthesis of proinflammatory cytokines and other molecules that are involved in inflammatory responses (45) (Figure 4A). These findings suggested that *T. canis* can cause inflammatory responses in puppy livers at the initial stage. At 24 hpi, the glycerolipid metabolism pathway, renin–angiotensin system pathway, and other types of O-glycan biosynthesis pathways were significantly enriched (Figure 4B). The glycerolipid metabolism pathway could perturb the host immune system and metabolism following anisakid infection (46). Further, the renin–angiotensin system pathway influences a range of processes from inflammation and immune responses to longevity (47). A previous study found that the O-glycoprotein biosynthesis pathway was associated with the immune response of dendritic cells (48). According to these findings, we assumed that *T. canis* larvae can trigger the immune responses in the infected host liver at 24 hpi. Some inflammation-related pathways were significantly enriched at 36 dpi, such as the cell adhesion molecules (CAMs) pathway, vitamin B6 metabolism pathway, cytokine–cytokine receptor interaction pathway, and glycosaminoglycan biosynthesis–keratan sulfate pathway (Figure 4C). The CAMs can direct mediate leukocyte migration, which is essential for generating effective inflammatory responses (49). Previous research found that vitamin B6 as a co-factor was involved in the inflammation response (50). It was reported that a high level of cytokine–cytokine motif chemokine ligand 1 could enhance and prolong the inflammatory response (51). The glycosaminoglycan pathway interacts with multiple ligands, which play an essential role in the inflammatory reaction (52). These results indicated that the puppies' livers may trigger inflammatory responses at the late stage of *T. canis* infection.

## Conclusion

The present study, for the first time, revealed the hepatic miRNA expression patterns of beagle dogs at three *T. canis* infection stages. A total of 59 DEmiRNAs were identified in the infected livers. The functional enrichment analysis of predicted target genes showed that miRNA cfa-miR-1839 could be related to the juvenile-to-adult transition of *T. canis* larvae. KEGG pathway analysis found that some significantly enriched pathways were related to the inflammatory response at 12 hpi and 36 dpi. Several miRNAs, such as novel-294, cfa-miR-88, cfa-miR-370, and cfa-miR-133c, were associated with immune responses. These findings should enrich our understanding of the interactions between *T. canis* and its definitive host. Further studies to elucidate the detailed molecular mechanisms and the physiological functions of the DEmiRNAs in the pathogenesis of *T. canis* infection are warranted.

## Data Availability Statement

The datasets presented in this study can be found in online repositories. The names of the repository/repositories and accession number(s) can be found at: https://www.ncbi.nlm.nih.gov/, PRJNA630302.

## Ethics Statement

The animal study was reviewed and approved by the Animal Administration and Ethics Committee of Lanzhou Veterinary Research Institute, Chinese Academy of Agricultural Sciences (Approval No. 2018-015). The dogs used in the study were handled in accordance with good animal practices required by the Animal Ethics Procedures and Guidelines of the People's Republic of China.

## Author Contributions

Y-XL and X-QZ conceived and designed the experiments. YZ and W-BZ performed the experiments. YZ analyzed the data and wrote the paper. J-JH and HME participated in improving the English of the manuscript. HME, X-QZ, and Y-XL critically revised the manuscript. All authors have read and approved the final version of the manuscript. All authors contributed to the preparation of the manuscript.

## Conflict of Interest

The authors declare that the research was conducted in the absence of any commercial or financial relationships that could be construed as a potential conflict of interest.
