# Supplementary material for: Toxocara canis Differentially Affects Hepatic MicroRNA Expression in Beagle Dogs at Different Stages of Infection
Source: Front Vet Sci. 2020 Nov 12;7:587273. doi: 10.3389/fvets.2020.587273 (PMC7689213; doi:10.3389/fvets.2020.587273)
Supplement: Supplementary file 2 [file Table_2.docx]

**Table S2.** The differently expressed miRNAs (*P* < 0.05) in puppy livers at different infection stages

| **Groups (post infection)** | **miRNA** | ***P*-vaule** | **Fold change** | **Expression** |
| --- | --- | --- | --- | --- |
| 12 hpi | novel_336 | 0.017464 | 1.80630 | Up-regulated |
|  | cfa-miR-371 | 0.018778 | 1.85912 | Up-regulated |
|  | novel_294 | 0.04126 | 1.61613 | Up-regulated |
|  | novel_318 | 0.043368 | 1.56009 | Up-regulated |
|  | cfa-miR-381 | 0.049294 | 1.60213 | Up-regulated |
|  | cfa-miR-10a | 0.033172 | 0.74802 | Down-regulated |
|  | cfa-miR-15b | 0.036681 | 0.79901 | Down-regulated |
|  | cfa-miR-885 | 0.041814 | 0.87742 | Down-regulated |
|  | cfa-miR-25 | 0.044349 | 0.80543 | Down-regulated |
| 24 hpi | cfa-miR-425 | 0.00018194 | 0.67587 | Down-regulated |
|  | cfa-miR-135a-5p | 0.0013155 | 1.08219 | Up-regulated |
|  | cfa-miR-125a | 0.0090894 | 1.34823 | Up-regulated |
|  | cfa-miR-214 | 0.019276 | 1.27740 | Up-regulated |
|  | cfa-miR-150 | 0.0196 | 1.32434 | Up-regulated |
|  | cfa-miR-497 | 0.024503 | 1.39317 | Up-regulated |
|  | cfa-miR-146a | 0.028156 | 0.69667 | Down-regulated |
|  | cfa-miR-10b | 0.029798 | 1.71029 | Up-regulated |
|  | cfa-miR-21 | 0.03196 | 0.73682 | Down-regulated |
|  | cfa-miR-1839 | 0.035675 | 0.78704 | Down-regulated |
|  | cfa-let-7g | 0.038544 | 0.83781 | Down-regulated |
|  | cfa-miR-7 | 0.040049 | 0.82777 | Down-regulated |
|  | cfa-miR-145 | 0.040795 | 1.35775 | Up-regulated |
|  | cfa-miR-26b | 0.046835 | 0.84208 | Down-regulated |
|  | cfa-miR-8884 | 0.048025 | 0.74466 | Down-regulated |
|  | cfa-miR-151 | 0.048029 | 1.30147 | Up-regulated |
| 36 dpi | cfa-miR-1 | 0.00063661 | 0.57094 | Down-regulated |
|  | cfa-miR-10b | 0.010022 | 0.64706 | Down-regulated |
|  | cfa-miR-127 | 0.0061135 | 0.53981 | Down-regulated |
|  | cfa-miR-129 | 0.021114 | 0.58046 | Down-regulated |
|  | cfa-miR-133c | 0.046275 | 0.65751 | Down-regulated |
|  | cfa-miR-136 | 0.012396 | 0.53683 | Down-regulated |
|  | cfa-miR-144 | 0.030744 | 1.45796 | Up-regulated |
|  | cfa-miR-146a | 0.048184 | 1.25860 | Up-regulated |
|  | cfa-miR-194 | 0.044603 | 1.16552 | Up-regulated |
|  | cfa-miR-205 | 0.012181 | 0.50082 | Down-regulated |
|  | cfa-miR-206 | 0.016733 | 0.61305 | Down-regulated |
|  | cfa-miR-223 | 0.0031789 | 0.55131 | Down-regulated |
|  | cfa-miR-23a | 0.0048681 | 1.66658 | Up-regulated |
|  | cfa-miR-23b | 0.021416 | 1.56137 | Up-regulated |
|  | cfa-miR-30a | 0.027407 | 0.88359 | Down-regulated |
|  | cfa-miR-335 | 0.048836 | 0.63253 | Down-regulated |
|  | cfa-miR-342 | 0.029497 | 1.37086 | Up-regulated |
|  | cfa-miR-370 | 0.020052 | 0.52217 | Down-regulated |
|  | cfa-miR-375 | 0.026045 | 0.77803 | Down-regulated |
|  | cfa-miR-379 | 6.18E-05 | 0.42635 | Down-regulated |
|  | cfa-miR-381 | 7.70E-05 | 0.51984 | Down-regulated |
|  | cfa-miR-382 | 0.0027283 | 0.56480 | Down-regulated |
|  | cfa-miR-411 | 0.001735 | 0.49938 | Down-regulated |
|  | cfa-miR-425 | 0.02143 | 0.63152 | Down-regulated |
|  | cfa-miR-433 | 0.039846 | 0.56149 | Down-regulated |
|  | cfa-miR-487b | 0.048241 | 0.57052 | Down-regulated |
|  | cfa-miR-495 | 0.028401 | 0.55596 | Down-regulated |
|  | cfa-miR-503 | 0.041481 | 1.35215 | Up-regulated |
|  | cfa-miR-676 | 0.046696 | 0.55773 | Down-regulated |
|  | cfa-miR-802 | 0.037183 | 1.49609 | Up-regulated |
|  | cfa-miR-889 | 0.0048083 | 0.48501 | Down-regulated |
|  | novel_103 | 0.0043606 | 0.54304 | Down-regulated |
|  | novel_180 | 0.033544 | 1.85890 | Up-regulated |
|  | novel_337 | 0.02841 | 1.90363 | Up-regulated |
